# The course of neuropsychiatric symptoms in nursing home residents from admission to 30-month follow-up

**DOI:** 10.1371/journal.pone.0206147

**Published:** 2018-10-18

**Authors:** Anne-Sofie Helvik, Geir Selbæk, Jūratė Šaltytė Benth, Irene Røen, Sverre Bergh

**Affiliations:** 1 General Practice Research Unit, Department of Public Health and Nursing, Faculty of Medicine and health Sciences, Norwegian University of Science and Technology (NTNU), Trondheim, Norway; 2 Norwegian National Advisory Unit on Ageing and Health, Vestfold Health Trust, Tønsberg, Norway; 3 St Olavs University Hospital, Trondheim, Norway; 4 Centre for Old Age Psychiatric Research, Innlandet Hospital Trust, Ottestad, Norway; 5 Institute of Health and Society, Faculty of Medicine, University of Oslo, Oslo, Norway; 6 Institute of Clinical Medicine, Campus Ahus, University of Oslo, Oslo, Norway; 7 HØKH, Research Centre, Akershus University Hospital, Lørenskog, Norway; University of Toronto, CANADA

## Abstract

**Aim:**

The aim of this study was to describe the prevalence and persistence of clinically significant neuropsychiatric symptoms (NPS) in nursing home residents with dementia, and to study the association between severity of dementia and specific neuropsychiatric sub-syndromes over time.

**Methods:**

In total, 583 residents with dementia were included at admission to a nursing home and followed with biannual assessments until death, or to 30-month follow-up. At the end of the 30-month follow-up, 305 participants had died and 57 had left the study for other reasons, leaving 221 residents in the study. We collected data on demographics, cognition, severity of dementia, NPS, personal activities of daily living (P-ADL), physical health, medication and type of nursing home unit. NPS was assessed using the Neuropsychiatric Inventory (NPI), the Nursing Home version.

**Results:**

The prevalence and persistence at two consecutive time-points of clinically significant NPS was high during the study period. The mean NPI agitation sub-syndrome score increased during the study period, while the NPI affective and psychosis sub-syndrome scores remained unchanged. More severe dementia was associated with higher NPI agitation, psychosis and affective sub-syndrome scores. The association remained unchanged over time for agitation and psychosis. For the NPI affective sub-syndrome, the association was stronger at the beginning, and declined towards the end of the study period.

**Conclusion:**

The findings of high prevalence and persistence at two consecutive time points of clinically significant NPS over time, and the associations between severity of dementia and NPI sub-syndromes shed light on the burden and care needs of nursing home residents with dementia after admission to nursing home care. This information is of interest to health care planners and providers to enable them to increase the quality of care for nursing home residents.

## Introduction

Neuropsychiatric symptoms (NPS) normally occur during the natural course of dementia [1]. NPS, include symptoms such as delusions, hallucination, depression, anxiety, euphoria, agitation, aggression, apathy and disinhibition.

NPS may either be subjectively experienced by the individual with dementia or observed by their next of kin or caregivers. In people with dementia, these symptoms may contribute to a feeling of distress and discomfort, and are associated with a poorer quality of life [2–5]. Furthermore, more severe NPS have been found to be associated with an accelerated cognitive decline [6, 7] and increased mortality [8], but the results regarding increased mortality are inconsistent [9]. NPS tend to increase depression [10] and reduce quality of life [11] for next of kin. Moreover, higher levels of NPS are associated with a greater burden on informal caregivers [10, 12] and an increase in the cost of care [13]. NPS are a cause of institutionalization for people with dementia [9, 14] and present a significant challenge in dementia care [15].

In Norway, as in other Western countries, a large proportion of nursing home residents suffer from dementia [16–20], and NPS are highly prevalent [21]. NPS show a heterogeneous course [22]. A review from 2013 based on seven follow-up studies of nursing home residents with dementia found that the persistence of individual NPS varied substantially, but most studies reported residents with at least one NPS [21]. One limitation of this review was that it included few studies and those studies featured a great deal of methodological diversity. For example, five different assessment tools were used to assess NPS, three of seven studies had fewer than 100 participants, and only two studies followed residents consecutively after admission to the nursing home. The first months after admission to a nursing home may be stressful for patients with dementia and accompanied with NPS. Thus, the length of nursing home stay prior to study inclusion may influence the prevalence and course of NPS described in the study [22]. Additionally, six of the seven studies had a follow-up of less than two years, and the time between assessments varied from 2 months to one year. Time between assessments and duration of follow-up may be important in determining the degree of persistence and associations reported [23]. Recently, a study of nursing home residents with dementia with four assessments over 53 months found agitation, irritability, disinhibition and apathy most prevalent and persistent during the study period, while an increase in dementia severity was associated with an increase in agitation, psychosis and apathy, but not affective symptoms [24]. There is a great need for new studies to further improve the knowledge concerning persistence and natural course of a broad spectrum of NPS in nursing home residents, and to address the methodological limitations reported [21–23]. In other words, we need large longitudinal studies that include residents consecutively at admission to a nursing home and where regular assessments over a longer study period are made. This information is vital for planning interventions and treatment of NPS in nursing home residents with dementia [21, 23].

The relationship between NPS, the dementia itself and individual and environmental factors is not fully understood [15, 25]. NPS might be an expression of the underlying brain disease [26], but increased vulnerability to stress and stimuli in the environment because of the disease may contribute to increasing the risk of NPS [25, 27][28]. In nursing home residents, environmental factors that contribute to stress and NPS may be characteristics of the psychosocial/physical environment, including type and degree of assistance received [15, 25, 29–31]. Apart from dementia, individual risks may include age, gender, marital status, physical functioning, physical health and use of medication [15, 23, 25].

The aims of this study were to describe the prevalence, incidence and persistence of clinically significant NPS in nursing home residents with dementia at admission and with biannual assessments over a 30-month follow-up period, and to study the association between the severity of dementia and the development of neuropsychiatric sub-syndromes using the Neuropsychiatric Inventory (NPI).

## Methods

### Design

This was an observational longitudinal study composed of participants from a convenience sample of 47 nursing homes in four Norwegian counties, representing small and large nursing homes that were located in both urban and rural areas [32]. The baseline data were collected within one month after admission to the nursing home between March 2012 and November 2014. The follow-up data were collected every six months or until the death of the participant. Follow-up assessments are still ongoing, but the present study includes information from baseline (T_1_) until the 30-month follow-up (T_6_), which all remaining participants had passed in May 2017.

### Setting and participants

With a population of about 5.3 million and about 700,000 (14%) people aged 65 years or older [33], Norway has about 40,000 nursing home places (beds) [34]. The country’s health care services are public, and jurisdiction lies within the local municipalities. Services provided include social services (such as housing and home services), in-home nursing and institutional care (mainly in nursing homes), and both long- and short-term care and rehabilitation.

In total, 696 residents with an expected stay longer than four weeks were recruited at admission to the nursing home. All residents 65 years and older independent of whether they had established dementia or not and residents younger than 65 years with established dementia were recruited at admission. The only exclusion criterion was a life expectancy of less than six weeks [32]. In the present article only those with dementia at admission were included. Two physicians (SB & GS) independently diagnosed dementia at baseline according to the ICD-10 criteria using all the available information at first assessment. In situations where the physicians disagreed, a third physician was consulted [32]. All physicians had extensive experience in research and clinical old age psychiatry. At admission to the nursing home, 583 residents had dementia and 113 residents did not have dementia [32]. Additional information regarding the setting and participants has been published elsewhere [32].

### Measures

Neuropsychiatric symptoms (NPS) were measured at each assessment using the Neuropsychiatric Inventory Nursing Home version (NPI-NH) [35]. The NPI is translated to Norwegian and validated [36]. The 12-item inventory were used, and it covers the following symptoms: delusion, hallucination, euphoria, agitation/aggression, disinhibition, irritability/lability, depression/dysphoria, anxiety, apathy/indifference, aberrant motor behavior, night-time behavior disturbances, and appetite and eating disorders (yes/no). Each symptom provides a score from zero to 12, i.e. severity (score 1–3) was multiplied by frequency (score 1–4). A score of four and higher was defined as a clinically significant symptom [37]. Three sub-syndrome scores were established, based on a previous principal component analysis, i.e. psychosis (including the sum-score of delusions and hallucination), agitation (including the sum-score of agitation/aggression, disinhibition and irritability), and affective (including the sum-score of depression and anxiety) [38].

Severity of dementia was measured at each assessment using the Norwegian version of the Clinical Dementia Rating (CDR) scale with five response categories (0, 0.5, 1, 2, 3) [39, 40]. The scale covers six domains (memory, orientation, judgment and problem solving, community affairs, home and hobbies and personal care), and the categorical score is calculated using an algorithm that gives priority to memory [39]. The categorical scores indicate dementia, ranging from 0 (no dementia) to 3 (severe dementia). A sum-score of the six domains (CDR Sum of Boxes, CDR-SoB), ranging from zero to 18 offers important advantages when analyzing data. A higher score is indicating more severe dementia. The correlation between the categorical CDR and the CDR-SoB is high [41, 42]. The Spearman correlation between the categorical CDR and the CDR-SoB score in the present study was 0.87 at baseline.

Use of psychotropic medications were collected from the medical record of each resident [43]. The medication were grouped according to the ATC code into the following categories: antipsychotics (N05A except lithium), antidepressants (N06A), anxiolytics (N05B), hypnotics/sedatives (N05C), and anti-dementia medication (N06D) (yes/no) [44].

Personal Activities of Daily Living (P-ADL) was assessed with the Physical Self-Maintenance Scale (PSMS) [45]. The scale includes six items with a total score ranging from 6 to 30 and higher scores indicate a lower level of functioning.

Physical health was assessed using a one-item global rating scale, i.e. the General Medical Health Rating (GMHR) scale [46]. The scale has four responses categories: very good, good, fairly good and poor. The rating was based on all available information of physical health and prescription drug use. The scale is previous used in large studies including older people with and without dementia [47] also in Norway [48].

Demographic information such as age, gender, and marital status was collected from the medical records. The type of unit was categorized as: regular unit (RU), and special care unit for people with dementia (SCU).

### Procedure

The data collection was undertaken by healthcare workers, mainly registered nurses (74%) in the nursing homes, under the supervision of 10 research nurses. The research nurses completed a five-day training program, while data collectors took a two-day training program prior to the data collection. The data came from a standardized interview with the residents, the next of kin, the residents’ caregivers in the nursing home, and from medical records.

The residents’ capacity to consent to participate in the study was assessed by the nursing home staff, including the nursing home physician. A written consent was obtained from all residents who had the capacity to give consent. If a resident lacked the ability to give consent, the resident’s next of kin consented on behalf of the resident. These procedures have been recommended and approved by the Norwegian Regional Ethics Committee South East (2011/1738a) [32].

### Data analysis

Sample characteristics at baseline were presented as means and standard deviations (SD) or frequencies and percentages. Prevalence, incidence and persistence in clinically significant NPS were calculated. Prevalence was defined as the proportion of nursing home residents with a specific clinically significant symptom present at each assessment; incidence was defined as the proportion of NH residents with a specific clinically significant symptom occurring for the first time at one assessment of the NH residents without the same clinically significant symptom reported from previous assessments; and persistence at two consecutive time points was defined as the proportion of NH residents with a specific clinically significant symptom at one assessment given the number of residents with the same clinically significant symptom at the previous assessments.

Time trends in sub-syndromes and NPI total scores were assessed by a linear mixed model with random effects for patients and units, and fixed effects for time up to second order. Next, CDR-SoB was included as a fixed effect along with the interaction between CDR-SoB and time. A significant interaction would imply a varying association between sub-syndromes and CDR-SoB throughout the follow-up period. Finally, models were adjusted for clinical and demographic characteristics. To easy the interpretation, the significant interactions were illustrated graphically for four arbitrarily chosen CDR-SoB values.

All analyses were performed in SPSS version 25 and SAS v9.4. Results with p-values below 0.05 were considered statistically significant. All tests were two-sided.

## Results

### Sample characteristics

At baseline, the mean (SD) age of the participants was 84.0 (7.5) years and 375 (64.3%) were women (Table 1). The mean (SD) baseline CDR-SoB was 11.2 (3.6).

**Table 1:** Sample characteristics at baseline (N = 583).

Characteristics			N
*Socio-demographics*			
Age	Mean (SD)	84.0 (7.5)	580
Females	N (%)	375 (64.3)	583
Married	N (%)	186 (32.3)	576
*Health condition*			
CDR-SoB	Mean (SD)	11.24 (3.59)	576
GMHR			
Fairly poor/Poor	N (%)	280 (50.3)	557
Good/Fairly good	N (%	277 (49.7	
PSMS score	Mean (SD)	15.3 (4.5)	582
Use of Psychotropic drugs			
Antipsychotics	N (%)	72 (12.3)	583
Antidepressants	N (%)	167 (28.6)	583
Anxiolytics	N (%)	89 (15.3)	583
Sedatives	N (%)	128 (22.0)	583
Anti-dementia drugs	N (%)	163 (28.0)	583
*NH characteristics*			
RU	N (%)	367 (63.0)	583
SCU	N (%)	216 (37.0)	
*Type of dementia*			
AD	N (%)	414 (71.0)	583
VAD	N (%)	46 (7.9)	
AD/VAD	N (%)	11 (1.9)	
FTD	N (%)	47 (8.1)	
LBD/PD	N (%)	22 (3.8)	
Unspecified	N (%)	43 (7.4)	

CDR-SoB: The sum-score of the domains in the Clinical Dementia Rating scale, GMHR: General Medical Health rating, PSMS: Physical Self-Maintenance Scale N H: Nursing home, RU: regular unit, SCU: Special care unit, AD: Alzheimer’s disease, VAD: Vascular dementia, AD/VAD: Alzheimer’s disease mixed type, FTD: Frontotemporal dementia, LBD/PD: Lewy body dementia/ Parkinson’s disease

Of the 583 residents at baseline, 210 (36%) were assessed after 30 months (Table 2). Attrition was mainly due to death (n = 305). Mean (SD) time of follow-up was 655.6 (305.6) days.

**Table 2:** Number of participants at each assessment in the study sample.

	T_1_ (Baseline)	T_2_ (6 months)	T_3_ (12 months)	T_4_ (18 months)	T_5_ (24 months)	T_6_ (30 months)
Number included	583	469	387	322	269	221
Number assessed	583	437	374	307	261	210
Number left		114	82	65	53	48
Due to death		84	67	61	51	42
Other reasons		30	15	4	2	6
NH withdrawn		1		2		
Patient withdrawn		4	2			
Moved to another unit of NH		13	5	2	1	5
Moved home		12	8		1	
Unknown						1

NH: Nursing home

### Prevalence and incidence of clinically significant neuropsychiatric symptoms over time

The prevalence of clinically significant NPS at baseline and at follow-up is presented in Table 3. The three most prevalent clinically significant NPS at baseline were depression (21.8%), anxiety (21.9%) and irritability (19.2%). The prevalence during follow-up varied between 21.3% to 23.2%, 20.8% to 26.5, and 24.8% to 37.1% for depression, anxiety and irritability, respectively. The least frequent clinically significant NPS at baseline were euphoria (3.7%) and hallucination (5.8%) and the prevalence of these symptoms was also low during the follow-up, between 4.2% to 6.4% and 5.0% to 10.4%, respectively. At baseline, 61.9% had at least one clinically significant NPS. The cumulative incidence of any clinically significant NPS thereafter was 28.7%. The cumulative incidence of clinically significant individual NPS was higher than 20% for eight of twelve symptoms (delusion, agitation, depression, anxiety, apathy, disinhibition, irritability and appetite and eating disorders), and lower than 10% for only one symptom, euphoria.

**Table 3:** Prevalence, incidence and persistence of significant neuropsychiatric symptoms (NPS) at each assessment (%).

	T_1_ (Baseline) N = 583	T_2_ (6 months) N = 437	T_3_ (12 months) N = 374	T_4_ (18 months) N = 307	T_5_ (24 months) N = 261	T_6_ (30 months) N = 210
Prevalence						
Delusions	16.0	18.5	19.9	22.3	22.5	21.7
Hallucinations	5.8	8.2	5.0	8.7	10.4	7.6
Agitation	16.2	16.0	21.6	22.3	27.1	25.1
Depression	21.8	22.0	21.6	23.2	21.3	21.6
Anxiety	21.9	20.8	21.2	24.9	26.5	24.1
Euphoria	3.7	4.2	4.6	5.3	6.4	5.9
Apathy	16.6	12.2	13.5	17.5	23.4	21.9
Disinhibition	16.1	16.6	18.5	19.0	29.2	26.6
Irritability	19.2	24.8	27.3	34.5	36.8	37.1
Aberrant Motor Behavior	12.0	11.4	12.1	14.4	15.1	14.0
Nighttime Behavior	17.0	15.0	11.8	12.0	13.9	10.3
Appetite and eating disorder	10.1	9.5	7.4	14.1	12.3	12.3
Any symptom	61.9	55.5	59.5	67.0	69.6	65.4
Cumulative incidence (%) and incidence between two consecutive time points						
	Cumulative incidence (N = 583)	T_1_-T_2_	T_2_-T_3_	T_3_-T_4_	T_4_-T_5_	T_5_-T_6_
Delusions	24.5	13.2	9.5	11.5	12.6	11.4
Hallucinations	10.8	5.8	2.0	4.5	3.7	2.4
Agitation	23.7	9.7	12.6	12.4	12.6	10.5
Depression	22.7	14.4	12.3	14.0	9.8	9.7
Anxiety	24.7	12.4	12.3	13.9	11.9	13.1
Euphoria	9.1	3.2	2.8	4.0	4.9	3.4
Apathy	25.5	7.8	10.4	12.1	15.7	9.7
Disinhibition	24.7	9.0	10.4	11.4	18.0	13.5
Irritability	31.6	15.0	13.6	22.0	15.0	21.0
Aberrant Motor Behavior	17.4	6.7	6.1	9.2	10.0	7.5
Nighttime Behavior	23.6	8.9	5.8	12.4	36.5	24.1
Appetite and eating disorder	28.7	33.7	33.7	47.9	8.3	7.6
Any symptom	15.8	7.9	4.5	6.4	8.1	4.3
Persistence of symptoms at two consecutive time points (%)						
		T_1_-T_2_	T_2_-T_3_	T_3_-T_4_	T_4_-T_5_	T_5_-T_6_
Delusions		46.4	66.7	62.1	52.7	54.3
Hallucinations		48.0	48.0	66.7	75.0	55.6
Agitation		52.3	72.5	57.6	70.7	63.6
Depression		52.3	59.4	57.4	58.8	58.1
Anxiety		52.7	58.8	61.7	67.7	61.7
Euphoria		27.8	31.3	33.3	35.7	54.5
Apathy		35.9	42.9	52.5	51.2	56.5
Disinhibition		53.7	58.2	56.9	63.3	63.0
Irritability		64.1	71.6	67.5	73.6	71.6
Aberrant Motor Behavior		43.4	57.1	56.3	38.9	50.0
Nighttime Behavior		51.4	50.0	45.9	64.0	44.8
Appetite and eating disorder		17.1	40.0	31.8	39.4	45.0
Any symptom		70.1	82.1	80.2	86.3	84.1

### Persistence of clinically significant NPS over time

The persistence of any clinically significant NPS was 70.1% between the two first assessments (baseline and 6-month follow-up), while the persistence of any clinically significant NPS for the remainder of the assessments varied between 80.2%-86.3%. The persistence of individual clinically significant NPS from baseline to the first follow-up was higher than 50% for agitation (52.3%), depression (52.3%), anxiety (52.7%), disinhibition (53.7%), irritability (64.1%) and nighttime behavior (51.4%). For these symptoms, the persistence during the rest of the study period was also higher than 50%, except for nighttime behavior. The persistence of irritability was higher than 70% three times during the study period (T_2_-T_3_, T_4_-T_5_ and T_5_-T_6_). The two symptoms with lowest persistence from baseline to the first follow-up were appetite and eating disorders (17.1%) and euphoria (27.8%). In the follow-up period, the persistence of these clinically significant NPS exceeded 30% from T_2_-T_3_ and remained at this level or higher throughout the study period. The persistence of apathy increased from 35.9% between the two first assessments (T_1_-T_2_) to 56.5% between the last two assessments (T_5_-T_6_).

### Factors associated with NPI sub-syndromes

**Fig 1:**
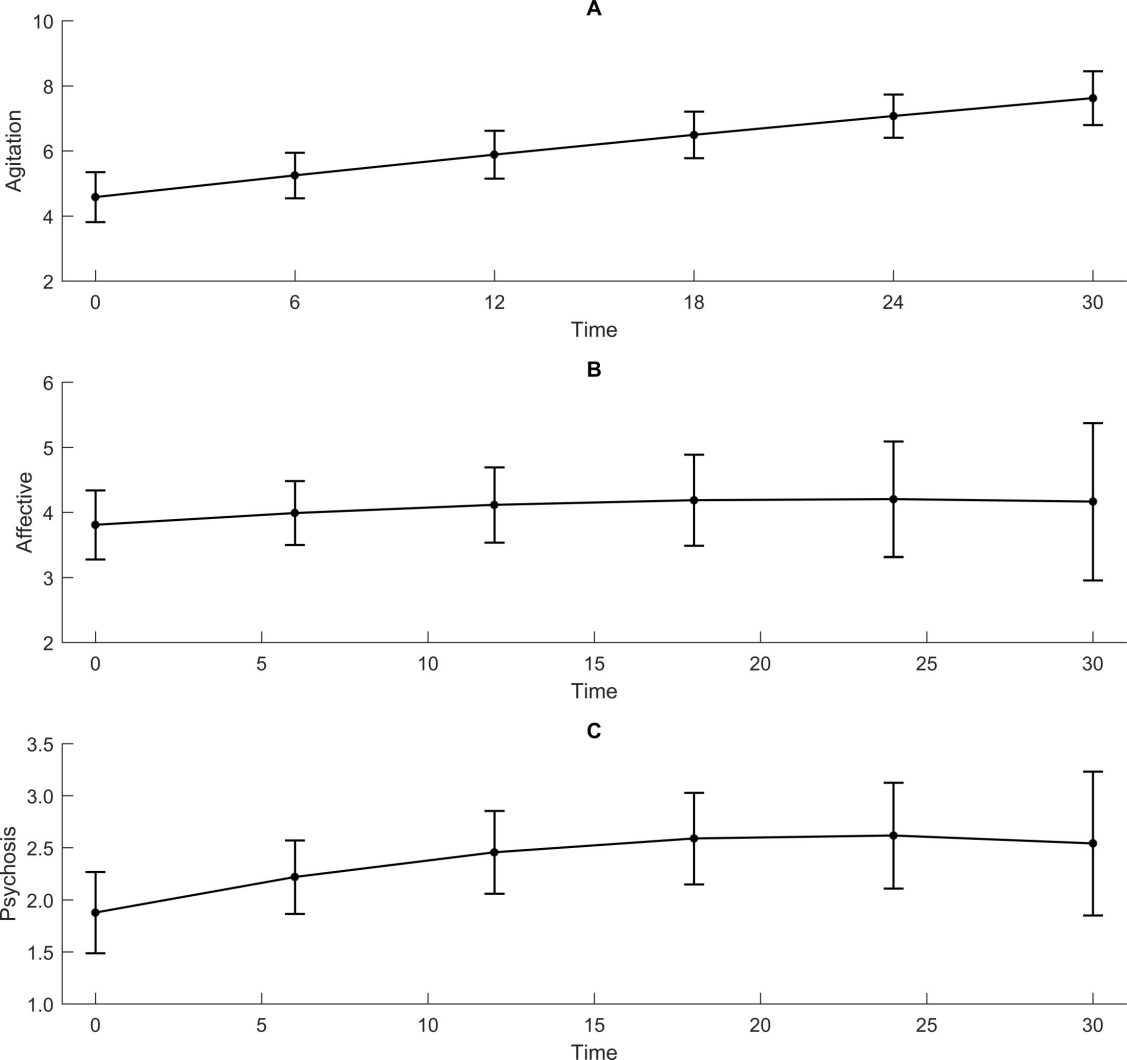
The mean (95% CI) NPI sub-syndrome scores over time for agitation (1A), affective (1B) and psychosis (1C).

**Fig 2:**
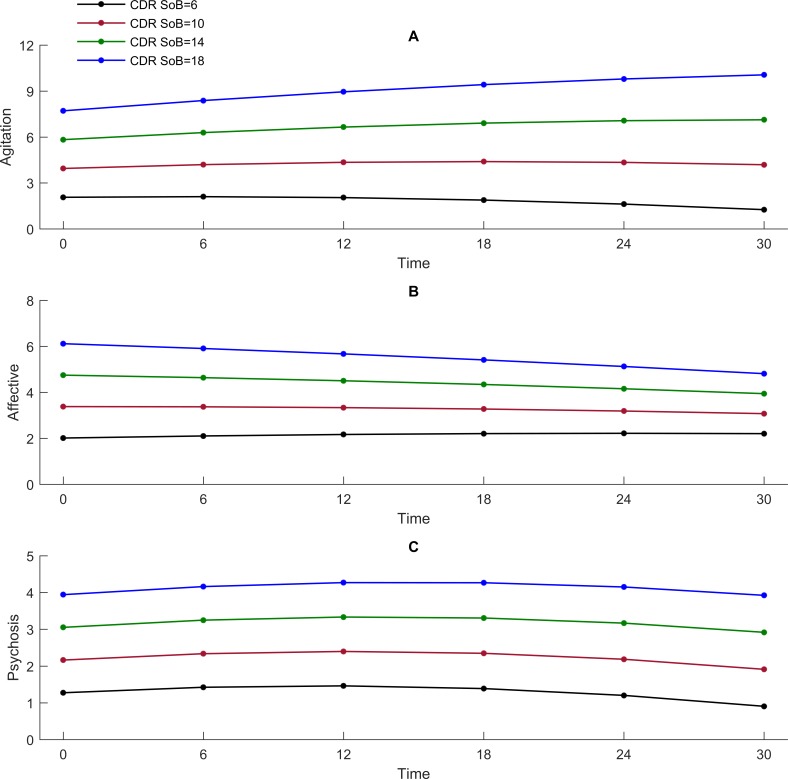
**The unadjusted associations of the NPI agitation sub-syndrome (2A), NPI affective sub-syndrome (2B) and NPI psychosis sub-syndrome (2C) by CDR-SoB over time**.

The mean NPI agitation sub-syndrome score increased significantly during the study period (Table 4 and Fig 1A). In an unadjusted analysis, higher CDR-SoB values were significantly associated with a higher NPI agitation sub-syndrome score measured simultaneously, with a stronger association towards the end of study period (Table 5 and Fig 2A). The increase in the NPI agitation sub-syndrome score throughout the study period was only significant for values of CDR-SoB of 8 or more.

**Table 4:** Sub-syndrome and total sum-scores of neuropsychiatric inventory at each time point.

	T_1_	T_2_	T_3_	T_4_	T_5_	T_6_
	(baseline)	(6 months)	(12 months)	(18 months)	(24 months)	(30 months)
NPI Agitation sub-syndrome						
N	561	423	361	298	249	198
Mean (SD)	4.5 (7.3)	5.0 (7.8)	5.8 (8.3)	6.0 (8.0)	7.9 (9.6)	7.2 (9.2)
NPI Psychosis sub-syndrome						
N	560	421	355	291	248	196
Mean (SD)	1.9 (4.2)	2.4 (4.6)	2.2 (3.9)	2.8 (4.7)	2.8 (4.7)	2.5 (4.0)
NPI Affective sub-syndrome						
N	572	429	364	296	245	198
Mean (SD)	3.9 (5.9)	3.8 (5.8)	3.7 (5.4)	4.3 (5.8)	4.0 (5.4)	3.7 (5.4)
NPI total sum-score						
N	535	402	335	270	233	186
Mean (SD)	14.9 (17.3)	15.3 (18.9)	15.7 (17.9)	17.5 (18.4)	19.7 (21.0)	18.7 (20.6)

NPI: Neuropsychiatric Inventory

After adjustment for demographic and clinical characteristics, the association between CDR-SoB and the NPI agitation sub-syndrome score remained significant throughout the follow-up. The NPI agitation sub-syndrome score significantly increased through the study period for CDR-SoB values of 12 or higher. In the same adjusted analysis, higher PSMS, use of antipsychotics and sedatives, lower age and staying in SCU at baseline were associated with higher NPI agitation sub-syndrome scores.

The mean NPI affective sub-syndrome score was stable during the study period (Table 4 and Fig 1B). In an unadjusted analysis, a higher CDR-SoB was associated with a higher NPI affective sub-syndrome score measured simultaneously, but the association became weaker with time (Table 5 and Fig 2B).

**Table 5:** Sub-syndromes of neuropsychiatric inventory by time and severity of dementia.

Variables	NPI Agitation				NPI Affective				NPI Psychosis			
	Unadjusted		Adjusted		Unadjusted		Adjusted		Unadjusted		Adjusted	
	Coeff (SE / 95% CI)	p-value	Coeff (SE / 95% CI)	p-value	Coeff (SE / 95% CI)	p-value	Coeff (SE / 95% CI)	p-value	Coeff (SE / 95% CI)	p-value	Coeff (SE / 95% CI)	p-value
Main variables												
Time	-0.04 (0.06)	0.542	-0.06 (0.06)	0.360	0.04 (0.04)	0.339	0.01 (0.04)	0.772	0.03 (0.03)	0.407	0.01 (0.03)	0.670
Time x Time	-0.001 (0.002)	0.386	-0.001 (0.002)	0.675	-0.0004 (0.001)	0.757	0.0006 (0.001)	0.597	-0.002 (0.0009)	0.086	-0.001 (0.001)	0.185
CDR-SoB	0.47 (0.07)	**<0.001**	0.36 (0.07)	**<0.001**	0.34 (0.05)	**<0.001**	0.23 (0.05)	**<0.001**	0.22 (0.04)	**<0.001**	0.21 (0.04)	**<0.001**
Time x CDR- SoB	0.009 (0.004)	**0.037**	0.007 (0.004)	0.084	-0.004 (0.003)	0.168	-0.006 (0.003)	**0.049**	0.001 (0.002)	0.677	0.001 (0.002)	0.565
Variables assessed at the same time point												
GMHR												
Good/Fairly good	0	-	0	-	0	-	0	-	0	-	0	-
Poor/Fairly bad	0.96 (0.26;1.66)	**0.007**	0.16 (-0.55;0.87)	0.662	0.98 (0.47;1.48)	**<0.001**	0.61 (0.09;1.12)	**0.020**	0.35 (-0.03;0.74)	0.072	0.20 (-0.20;0.59)	0.330
Marital status												
Not married	-1.77 (-2.86;-0.69)	**0.001**	-0.77 (-1.83;0.29)	0.155	-0.57 (-1.36;0.22)	0.160	-0.45 (-1.24;0.34)	0.266	-0.88 (-1.47; -0.30)	**0.003**	-0.74 (-1.32;-0.15)	**0.014**
Married	0	-	0	-	0	-	0	-	0	-	0	-
PSMS score	0.35 (0.27;0.44)	**<0.001**	0.14 (0.04;0.24)	**0.007**	0.23 (0.17; 0.30)	**<0.001**	0.13 (0.06;0.21)	**0.001**	0.09 (0.04;0.13)	**<0.001**	-0.03 (-0.08;0.03)	0.361
Use of PTD												
Antipsychotics	2.53 (1.51;3.56)	**<0.001**	1.81 (0.82;2.81)	**<0.001**	1.55 (0.81;2.30)	**<0.001**	1.04 (0.31;1.77)	**0.005**	1.90 (1.33;2.46)	**<0.001**	1.61 (1.05;2.17)	**<0.001**
Antidepressants	0.45 (-0.38;1.28)	0.288	-0.03 (-0.84;0.77)	0.941	1.92 (1.33;2.51)	**<0.001**	1.57 (0.99; 2.16)	**<0.001**	0.13 (-0.32;0.58)	0.580	-0.11 (-0.56;0.34)	0.625
Anxiolytics	1.02 (0.09;1.95)	**0.032**	0.67 (-0.23; 1.58)	0.143	0.61 (-0.06;1.28)	0.077	0.17 (-0.49;0.82)	0.618	0.69 (0.18;1.20)	**0.008**	0.47 (-0.03;0.98)	0.066
Sedatives	1.54 (0.69;2.39)	**<0.001**	1.53 (0.70;2.36)	**<0.001**	1.24 (0.63; 1.88)	**<0.001**	0.96 (0.36;1.56)	**0.002**	0.70 (0.23;1.16)	**0.003**	0.57 (0.11;1.03)	**0.016**
Variables assessed at baseline												
Age	-0.19 (-0.26;-0.12)	**<0.001**	-0.14 (-0.21;-0.06)	**<0.001**	-0.07 (-0.12;-0.01)	**0.016**-	-0.03 (-0.09;0.02)	0.232	-0.03 (-0.08;0.005)	0.089	-0.007 (-0.05;0.03)	0.718
Gender												
Females	-1.05 (-2.18;0.09	0.072	-0.34 (-1.41;0.73)	0.534	1.22 (0.40;2.05)	**0.004**	1.45 (0.65;2.26)	**<0.001**	-0.12 (-0.72;0.49)	0.707	0.17 (-0.42;0.76)	0.576
Males	0	**-**	0	**-**	0	**-**	0	-	0	-	0	-
Type NH unit												
SCU	3.28 (2.12;4.45)	**<0.001**	2.17 (1.14;3.20)	**<0.001**	1.12 (0.27;1.96)	**0.010**	0.81 (-0.02;1.63)	0.055	1.29 (0.69;1.88)	**<0.001**	0.79 (0.22;1.35)	**0.007**
RU	0	**-**	0	**-**	0	**-**	0	-	0	-	0	-

CDR-SoB: Clinical Dementia Rating—Sum of Boxes, GMHR: General Medical Health rating, PSMS: Physical Self-Maintenance Scale. PTD: Psychotropic medications, NH: Nursing home, RU: Regular unit, SCU: Special care unit

In the adjusted analysis, a higher CDR-SoB was associated with a higher NPI affective sub-syndrome score in the beginning of the study period, but the strength of the association diminished at the end of the study period. In the same adjusted analysis, poor GMHR, higher PSMS, female gender, and use of antipsychotics, antidepressants and sedatives were associated with a higher NPI affective sub-syndrome score.

The mean NPI psychosis sub-syndrome score was quite stable during the study period (Table 4 and Fig 1C). In both unadjusted and adjusted analyses, a higher CDR-SoB score was associated with a higher score on the NPI psychosis sub-syndrome score measured simultaneously, and the association remained stable for all values of CDR-SoB (Table 5 and Fig 2C). In the adjusted model, being married, use of antipsychotics and sedatives, and being a resident in SCU at baseline were associated with a higher NPI psychosis sub-syndrome score.

## Discussion

The prevalence and persistence of at least one clinically significant NPS was high from admission to NH and throughout the 30-month study period, i.e. ranging from 56% to 70% and 70% to 86%, respectively. The three most prevalent NPS at baseline were irritability, depression, and anxiety, and the prevalence of these symptoms remained high. The persistence of irritability, depression, anxiety, agitation and disinhibition exceeded 50% between all assessments. The mean NPI agitation sub-syndrome score increased during the study period, while the mean NPI affective and psychosis sub-syndrome scores remained unchanged. More severe dementia was associated with a higher NPI agitation and psychosis sub-syndrome score, an association that remained unchanged over time. More severe dementia was associated with a higher score on the NPI affective sub-syndrome in the beginning of the study period, but the strength of the association fell over time, and at the end of the study period the association had nearly disappeared. Use of antipsychotics and sedatives were associated with higher scores of all NPI sub-syndromes measured at the same time point.

The high prevalence and persistence between two consecutive assessments of at least one clinically significant NPS found in the present study is in line with previous studies [24]. The cumulative incidence of any clinical significant NPS after baseline was 29%. The high baseline prevalence (61.9%) along with the cumulative incidence indicate that most residents with dementia had at least one NPS at one time point during the study period [1].

Agitation is a clinical concept that includes a number of different symptoms [24]. A review of studies using NPI in persons with Alzheimer’s disease revealed that the symptoms included in this sub-syndrome may vary [49]. Even so, the review did not include nursing home studies. In Norwegian studies of nursing home residents with dementia, the items agitation/aggression, disinhibition and irritability have been used to comprise the NPI agitation sub-syndrome, which is comparable to the symptoms used in international nursing home studies of residents with dementia [38, 50–52]. In the present study, the high baseline prevalence of the clinically significant symptom of irritability increased over time, as did the clinically significant symptoms of agitation and disinhibition. Furthermore, the persistence was high for these clinically significant symptoms at all consecutively compared time points. Our findings on prevalence and persistence were similar to results from studies that did not consecutively include nursing home residents with dementia at admission [22, 24]. The high and over time increasing prevalence as well as the high persistence of these symptoms in the present study and the previous [22, 24] underscore the strain the nursing home residents may experience, but also the care challenges the nursing staff may experience related to these symptoms. The mean score for the NPI agitation sub-syndrome increased during the entire study period of 30 months. There was an association between greater severity of dementia and a higher agitation score. The agitation score increased through the study period for those with more severe dementia (CDR-SoB ≥ 12). This finding is comparable with a previous small longitudinal study of nursing home residents with dementia over 18 months with biannual assessments [53]. Agitation in residents with the most severe dementia increased to last follow-up, but the level of agitation did not change over time in those with moderate dementia [53]. Agitation in residents with dementia has been linked to unmet needs [54]. The unmet needs model postulates that the dementia process decreases residents’ ability to satisfy or accommodate their needs [54]. Those with severe dementia have severely limited abilities to express their needs due to impaired communication and cognition. For care staff, identification of these patients’ needs is harder as the severity of the patient’s dementia increases, and even when recognized, the caregivers may not fulfill them [55]. In addition, greater agitation and more severe dementia are both linked to the underlying brain disease [26]. The increase in agitation over time may partly be a result of the advancing brain disease in residents with the most severe dementia. Knowledge about the course of the NPI agitation sub-syndrome over time in general, the relation between severity of dementia and the severity of agitation and the unmet needs model are important for health care planners and nursing staff so they can better organize and adapt care for residents. In addition, it is important for caregivers to keep in mind that poorer P-ADL function as well as younger age might be factors important in generating higher NPI agitation sub-syndrome scores. Several studies have found an association between younger age and more pronounced P-ADL impairment and higher agitation [56]. We found that the severity of agitation was higher among residents in SCU. One explanation, which is in keeping with our clinical experience, may be that severe NPS is a reason for admission to an SCU. However, due to the nature of this study, we cannot rule out the possibility that being in an SCU is an additional, exacerbating factor for the severity of agitation.

The NPI affective sub-syndrome in the present study included depression and anxiety symptoms. Clinically significant depression and anxiety were among the most frequent NPS registered at baseline in our study, with a prevalence level that was comparable to other studies [24]. The prevalence of clinically significant depression and anxiety and the mean score of the NPI affective sub-syndrome remained unchanged during the 30-month study period. The persistence of clinically significant symptoms of depression and anxiety at two consecutive time points was higher than 50%, and the cumulative incidence of these symptoms was considerable. This indicates an intermittent course of depression and anxiety symptoms, as has been found by others exploring NPS in nursing home residents with dementia [23, 25]. This finding merit further investigations. Unlike most nursing home studies, the present study followed residents with dementia from admission, which gave us the opportunity to explore the course of affective symptoms after admission. We found that residents with more severe dementia had a higher NPI affective sub-syndrome score in the first 18 months after admission to the nursing home compared to residents with less severe dementia, but also that the differences in the NPI affective sub-syndrome scores according to the severity of the dementia diminished over the study period. This may be explained by the fact that individuals with severe dementia are more vulnerable for stress and change in of environment [27, 28, 57, 58]. Even so, after some time, affective symptoms may be attenuated as residents learn to adjust to the situation [59]. Even if affective symptoms in dementia may be understood as ineffective attempts of the resident to cope with stress factors, other explanations may be found. It might be that residents with more severe dementia over time have more difficulties expressing affective symptoms than residents with less severe dementia due to more severe communication difficulties. Thus, it could be harder for caregivers to understand the patient’s expression of anxiety and depression, which could be mistaken for apathy. There is substantial evidence suggesting that affective symptoms share complex pathophysiological routes with dementia [25]. NPS generally tend to worsen when the severity of dementia increases [21, 56, 60]. Even so, not all studies have found an association between severity of dementia and severity of affective symptoms in nursing home residents, but only a minority of these studies have included residents beginning with admission to the nursing home [56] [21, 22, 61]. However, other studies have found an association between female gender, poor health, personal functioning and affective symptoms [56], as we found in our study, but the findings on the association between gender and affective symptoms are conflicting [29]. The impact of gender as a biologic variable in relation to challenges due to dementia remains elusive [62].

The NPI psychosis sub-syndrome in nursing home residents with dementia includes delusions and hallucination [38, 50–52]. The prevalence of clinically significant symptoms of delusion and hallucinations at baseline in the present study was 16 and 6%, respectively. The prevalence and cumulative incidence of clinical significant hallucinations and delusions in our study were comparable to findings in other longitudinal studies of nursing home residents with dementia [19, 21, 29, 36, 63]. The overall mean score of the NPI psychosis sub-syndrome was mostly unchanged during the 30-month follow-up. More severe dementia was associated with a higher NPI psychosis sub-syndrome score, with the association stable over time. In contrast to our study, a study that followed nursing home residents over 53 months reported that the psychosis sub-syndrome score declined somewhat in residents with moderate and severe dementia, but increased in residents with mild dementia [24]. The differences in these and our findings may be due to the differences in the length of follow-up, and how the studies handled the inclusion of residents with dementia. Like most other studies of nursing home residents, the 53-month follow-up study included residents with varying lengths of residency prior to study inclusion, while the present study included residents at admission to the nursing home.

We were surprised to find that both the use of antipsychotics and sedatives were associated with higher severity for all NPI sub-syndromes and that the use of antidepressants was associated with a higher severity for the NPI affective sub-syndrome. These findings could be considered counterintuitive. Even if it is known that the use of psychotropic medication may not have the expected effect on NPS [15] and could have considerable adverse effects [64], we do not know whether the association relates to a lack of benefits or adverse effects from the medication. It could also be that people taking psychotropic medication had had even more severe symptoms prior to starting the medication. Thus, the relationship between the use of psychotropic drugs and severity of NPS needs further attention, and our results should be interpreted with caution.

### Strength and limitations

The large sample of residents with dementia recruited at nursing home admission is one of the major strengths of our study, because it gave us the opportunity to perform robust analyses and to adjust for many potentially important variables. Secondly, our use of NPI to assess NPS is an important facet of the study, because NPI covers a broad spectrum of symptoms, has been extensively tested [36, 49, 65], is frequently used [23], and is recommended as the core tool in NPS assessments [23]. Our use of NPI also allows our study to be compared to other studies, because the tool is frequently used across samples or populations.

The study has some limitations. Firstly, inclusion in the study was not based on a random selection from all nursing homes in Norway’s 19 counties, and thus we cannot claim the sample is representative of Norwegian nursing home residents. However, this study included residents from 47 nursing homes located in four counties, and from both small and large nursing homes in urban and rural areas. Secondly, some residents were lost to follow-up due to the nursing home or the resident withdrew from the study (9), or the resident moved to another location, i.e. another nursing home unit (26) or home (21), or for unknown reasons (1). The relatively large number of dropouts is a problem inherent in most longitudinal nursing home studies, and may restrict generalization of the results. However, in the present study, we used statistical methods suitable for unbalanced data sets and included information on all participants at each assessment, including dropouts. Thirdly, we based the assessment of the severity of dementia on the CDR rating of several assessors. The CDR assessment was included in the standardized dementia diagnostic procedure. Even though all health care workers who undertook the data collection participated in a two-day educational course to secure adequate knowledge and high data quality, we cannot rule out the possibility that the large number of assessors could have biased the results. However, the data collection was done under the supervision of 10 research nurses who had attended a five-day training program prior to study start to reduce this risk. Lastly, even though we adjusted for several health and demographic variables in the analysis, pain, which can be a confounder, was not included.

## Conclusion

The prevalence and persistence at two consecutive time points of clinically significant NPS was high during the study period. The mean NPI agitation sub-syndrome score increased during the study period, while the mean NPI affective and psychosis sub-syndrome scores remained unchanged. More severe dementia was associated with higher NPI agitation, psychosis and affective sub-syndrome scores. The association remained unchanged over time for agitation and psychosis. For the NPI affective sub-syndrome, the association was stronger at the beginning of the study period, while over time the association related to the severity of dementia declined. These findings may contribute to improve the understanding of the burden and care needs that nursing home residents with dementia pose. Such knowledge is of importance both for health care planners and caregivers in order to increase the quality of care for nursing home residents.

## References

[1] Steinberg M, Shao H, Zandi P, Lyketsos CG, Welsh-Bohmer KA, Norton MC, et al Point and 5-year period prevalence of neuropsychiatric symptoms in dementia: the Cache County Study. Int J Geriatr Psychiatry. 2008;23(2):170–7. Epub 2007/07/04. 10.1002/gps.1858 ; PubMed Central PMCID: PMC2932652. 17607801 PMC2932652

[2] Wetzels RB, Zuidema SU, de Jonghe JF, Verhey FR, Koopmans RT. Determinants of quality of life in nursing home residents with dementia. Dement Geriatr Cogn Disord. 2010;29(3):189–97. Epub 2010/03/11. 10.1159/000280437 . 20215750

[3] van de Ven-Vakhteeva J, Bor H, Wetzels RB, Koopmans RT, Zuidema SU. The impact of antipsychotics and neuropsychiatric symptoms on the quality of life of people with dementia living in nursing homes. Int J Geriatr Psychiatry. 2013;28(5):530–8. Epub 2012/08/14. 10.1002/gps.3858 . 22886912

[4] Haaksma ML, Leoutsakos JS, Bremer JA, Aalten P, Ramakers IH, Verhey FR, et al The clinical course and interrelations of dementia related symptoms. Int Psychogeriatr. 2017:1–8. Epub 2017/03/14. 10.1017/s1041610217000321 . 28285610

[5] Hongisto K, Hallikainen I, Selander T, Tormalehto S, Vaatainen S, Martikainen J, et al Quality of Life in relation to neuropsychiatric symptoms in Alzheimer's disease: 5-year prospective ALSOVA cohort study. Int J Geriatr Psychiatry. 2017 Epub 2017/01/10. 10.1002/gps.4666 . 28067961

[6] Emanuel JE, Lopez OL, Houck PR, Becker JT, Weamer EA, Demichele-Sweet MA, et al Trajectory of cognitive decline as a predictor of psychosis in early Alzheimer disease in the cardiovascular health study. Am J Geriatr Psychiatry. 2011;19(2):160–8. Epub 2010/09/03. 10.1097/JGP.0b013e3181e446c8 ; PubMed Central PMCID: PMCPMC3000865. 20808116 PMC3000865

[7] Baker E, Iqbal E, Johnston C, Broadbent M, Shetty H, Stewart R, et al Trajectories of dementia-related cognitive decline in a large mental health records derived patient cohort. PloS one. 2017;12(6):e0178562 Epub 2017/06/08. 10.1371/journal.pone.0178562 ; PubMed Central PMCID: PMCPMC5462385. 28591196 PMC5462385

[8] Russ TC, Batty GD, Starr JM. Cognitive and behavioural predictors of survival in Alzheimer disease: results from a sample of treated patients in a tertiary-referral memory clinic. Int J Geriatr Psychiatry. 2012;27(8):844–53. Epub 2011/10/01. 10.1002/gps.2795 . 21956773

[9] Wergeland JN, Selbaek G, Bergh S, Soederhamn U, Kirkevold O. Predictors for Nursing Home Admission and Death among Community-Dwelling People 70 Years and Older Who Receive Domiciliary Care. Dementia and Geriatric Cognitive Disorders EXTRA. 2015;5(3):320–9. Epub 2015/10/21. 10.1159/000437382 ; PubMed Central PMCID: PMCPMC4608662. 26483831 PMC4608662

[10] Sink KM, Covinsky KE, Barnes DE, Newcomer RJ, Yaffe K. Caregiver characteristics are associated with neuropsychiatric symptoms of dementia. Journal of the American Geriatrics Society. 2006;54(5):796–803. Epub 2006/05/16. 10.1111/j.1532-5415.2006.00697.x . 16696746

[11] Lethin C, Renom-Guiteras A, Zwakhalen S, Soto-Martin M, Saks K, Zabalegui A, et al Psychological well-being over time among informal caregivers caring for persons with dementia living at home. Aging Ment Health. 2017;21(11):1138–46. Epub 2016/07/28. 10.1080/13607863.2016.1211621 . 27463390

[12] Lorenzo-Lopez L, de Labra C, Maseda A, Lorenzo T, Agrafojo H, Rodriguez-Villamil JL, et al Caregiver's distress related to the patient's neuropsychiatric symptoms as a function of the care-setting. Geriatric nursing (New York, NY). 2017;38(2):110–8. Epub 2016/09/14. 10.1016/j.gerinurse.2016.08.004 . 27623026

[13] Murman DL, Chen Q, Powell MC, Kuo SB, Bradley CJ, Colenda CC. The incremental direct costs associated with behavioral symptoms in AD. Neurology. 2002;59(11):1721–9. Epub 2002/12/11. . 12473759 10.1212/01.wnl.0000036904.73393.e4

[14] Gaugler JE, Yu F, Krichbaum K, Wyman JF. Predictors of nursing home admission for persons with dementia. Medical care. 2009;47(2):191–8. Epub 2009/01/27. 10.1097/MLR.0b013e31818457ce . 19169120

[15] Kales HC, Gitlin LN, Lyketsos CG. Management of neuropsychiatric symptoms of dementia in clinical settings: recommendations from a multidisciplinary expert panel. Journal of the American Geriatrics Society. 2014;62(4):762–9. Epub 2014/03/19. 10.1111/jgs.12730 ; PubMed Central PMCID: PMC4146407. 24635665 PMC4146407

[16] Seitz D, Purandare N, Conn D. Prevalence of psychiatric disorders among older adults in long-term care homes: a systematic review. Int Psychogeriatr. 2010;22(7):1025–39. Epub 2010/06/05. 10.1017/S1041610210000608 . 20522279

[17] Schaufele M, Kohler L, Hendlmeier I, Hoell A, Weyerer S. [Prevalence of dementia and medical care in German nursing homes: a nationally representative survey]. Psychiatr Prax. 2013;40(4):200–6. Epub 2013/05/15. 10.1055/s-0033-1343141 . 23670714

[18] Kowalska J, Rymaszewska J, Szczepanska-Gieracha J. Occurrence of cognitive impairment and depressive symptoms among the elderly in a nursing home facility. Adv Clin Exp Med. 2013;22(1):111–7. Epub 2013/03/08. . 23468269

[19] Bergh S, Engedal K, Roen I, Selbaek G. The course of neuropsychiatric symptoms in patients with dementia in Norwegian nursing homes. Int Psychogeriatr. 2011;23(8):1231–9. Epub 2011/07/07. 10.1017/S1041610211001177 . 21729409

[20] Bergh S, Holmen J, Saltvedt I, Tambs K, Selbaek G. Dementia and neuropsychiatric symptoms in nursing-home patients in Nord-Trondelag County. Tidsskr Nor Laegeforen. 2012;132(17):1956–9. Epub 2012/09/26. 10.4045/tidsskr.12.0194 . 23007358

[21] Selbaek G, Engedal K, Bergh S. The prevalence and course of neuropsychiatric symptoms in nursing home patients with dementia: a systematic review. Journal of the American Medical Directors Association. 2013;14(3):161–9. Epub 2012/11/22. 10.1016/j.jamda.2012.09.027 . 23168112

[22] Wetzels R, Zuidema S, Jansen I, Verhey F, Koopmans R. Course of neuropsychiatric symptoms in residents with dementia in long-term care institutions: a systematic review. Int Psychogeriatr. 2010;22(7):1040–53. Epub 2010/08/04. 10.1017/S1041610210000918 . 20678299

[23] van der Linde RM, Dening T, Stephan BC, Prina AM, Evans E, Brayne C. Longitudinal course of behavioural and psychological symptoms of dementia: systematic review. The British journal of psychiatry: the journal of mental science. 2016;209(5):366–77. Epub 2016/11/03. 10.1192/bjp.bp.114.148403 ; PubMed Central PMCID: PMCPMC5100633. 27491532 PMC5100633

[24] Selbaek G, Engedal K, Benth JS, Bergh S. The course of neuropsychiatric symptoms in nursing-home patients with dementia over a 53-month follow-up period. Int Psychogeriatr. 2014;26(1):81–91. Epub 2013/09/26. 10.1017/S1041610213001609 . 24059840

[25] Cerejeira J, Lagarto L, Mukaetova-Ladinska EB. Behavioral and psychological symptoms of dementia. Frontiers in neurology. 2012;3:73 Epub 2012/05/16. 10.3389/fneur.2012.00073 ; PubMed Central PMCID: PMCPMC3345875. 22586419 PMC3345875

[26] Van Dam D, Vermeiren Y, Dekker AD, Naude PJ, Deyn PP. Neuropsychiatric Disturbances in Alzheimer's Disease: What Have We Learned from Neuropathological Studies? Current Alzheimer research. 2016;13(10):1145–64. Epub 2016/05/04. 10.2174/1567205013666160502123607 ; PubMed Central PMCID: PMCPMC5070416. 27137218 PMC5070416

[27] Kales HC, Gitlin LN, Lyketsos CG. Assessment and management of behavioral and psychological symptoms of dementia. BMJ (Clinical research ed). 2015;350:h369 Epub 2015/03/04. 10.1136/bmj.h369 ; PubMed Central PMCID: PMCPMC4707529. 25731881 PMC4707529

[28] Tible OP, Riese F, Savaskan E, von Gunten A. Best practice in the management of behavioural and psychological symptoms of dementia. Therapeutic advances in neurological disorders. 2017;10(8):297–309. Epub 2017/08/07. 10.1177/1756285617712979 ; PubMed Central PMCID: PMCPMC5518961. 28781611 PMC5518961

[29] Brodaty H, Draper B, Saab D, Low LF, Richards V, Paton H, et al Psychosis, depression and behavioural disturbances in Sydney nursing home residents: prevalence and predictors. Int J Geriatr Psychiatry. 2001;16(5):504–12. Epub 2001/05/29. . 11376467 10.1002/gps.382

[30] Zuidema SU, de Jonghe JF, Verhey FR, Koopmans RT. Environmental correlates of neuropsychiatric symptoms in nursing home patients with dementia. Int J Geriatr Psychiatry. 2010;25(1):14–22. Epub 2009/06/12. 10.1002/gps.2292 . 19517419

[31] Kunik ME, Snow AL, Davila JA, Steele AB, Balasubramanyam V, Doody RS, et al Causes of aggressive behavior in patients with dementia. The Journal of clinical psychiatry. 2010;71(9):1145–52. Epub 2010/04/07. 10.4088/JCP.08m04703oli . 20361896

[32] Roen I, Selbaek G, Kirkevold O, Engedal K, Testad I, Bergh S. Resourse Use and Disease Couse in dementia—Nursing Home (REDIC-NH), a longitudinal cohort study; design and patient characteristics at admission to Norwegian nursing homes. BMC health services research. 2017;17(1):365 Epub 2017/05/24. 10.1186/s12913-017-2289-x ; PubMed Central PMCID: PMCPMC5441072. 28532443 PMC5441072

[33] Boltz M, Resnick B, Chippendale T, Galvin J. Testing a family-centered intervention to promote functional and cognitive recovery in hospitalized older adults. J Am Geriatr Soc. 2014;62(12):2398–407. Epub 2014/12/09. 10.1111/jgs.13139 . 25481973 PMC4883662

[34] Statsbudsjettet 2013 In: Finansdepartementet, editor. Oslo: http://www.statsbudsjettet.no/Statsbudsjettet-2013/Budsjettsporsmal/Bevilgningssporsmal/Hyre144/?all=true&parti=h%C3%B8yre2013.

[35] Cummings JL. The Neuropsychiatric Inventory: assessing psychopathology in dementia patients. Neurology. 1997;48(5 Suppl 6):S10-6. Epub 1997/05/01. . 9153155 10.1212/wnl.48.5_suppl_6.10s

[36] Selbaek G, Kirkevold O, Sommer OH, Engedal K. The reliability and validity of the Norwegian version of the Neuropsychiatric Inventory, nursing home version (NPI-NH). Int Psychogeriatr. 2008;20(2):375–82. Epub 2007/06/15. 10.1017/S1041610207005601 . 17559707

[37] Steinberg M, Tschanz JT, Corcoran C, Steffens DC, Norton MC, Lyketsos CG, et al The persistence of neuropsychiatric symptoms in dementia: the Cache County Study. Int J Geriatr Psychiatry. 2004;19(1):19–26. Epub 2004/01/13. 10.1002/gps.1025 . 14716695

[38] Selbaek G, Engedal K. Stability of the factor structure of the Neuropsychiatric Inventory in a 31-month follow-up study of a large sample of nursing-home patients with dementia. Int Psychogeriatr. 2012;24(1):62–73. Epub 2011/06/21. 10.1017/S104161021100086X . 21682940

[39] Hughes CP, Berg L, Danziger WL, Coben LA, Martin RL. A new clinical scale for the staging of dementia. Br J Psychiatry. 1982;140:566–72. . 7104545 10.1192/bjp.140.6.566

[40] Morris JC. The Clinical Dementia Rating (CDR): current version and scoring rules. Neurology. 1993;43(11):2412–4. . 8232972 10.1212/wnl.43.11.2412-a

[41] Mjorud M, Kirkevold M, Rosvik J, Selbaek G, Engedal K. Variables associated to quality of life among nursing home patients with dementia. Aging Ment Health. 2014:1–9. Epub 2014/06/10. 10.1080/13607863.2014.903468 . 24911813

[42] O'Bryant SE, Waring SC, Cullum CM, Hall J, Lacritz L, Massman PJ, et al Staging dementia using Clinical Dementia Rating Scale Sum of Boxes scores: a Texas Alzheimer's research consortium study. Arch Neurol. 2008;65(8):1091–5. Epub 2008/08/13. 10.1001/archneur.65.8.1091 ; PubMed Central PMCID: PMC3409562. 18695059 PMC3409562

[43] Selbaek G, Kirkevold O, Engedal K. The prevalence of psychiatric symptoms and behavioural disturbances and the use of psychotropic drugs in Norwegian nursing homes. Int J Geriatr Psychiatry. 2007;22(9):843–9. Epub 2006/12/29. 10.1002/gps.1749 . 17193341

[44] 2015 WCCfDSM. WHO Collaborating Centre for Drug Statistics Metodology ATC/DDD Index, 2015, Oslo: Noreweigan Institue of Public Health; 2015 [26.08.2015].

[45] Lawton MP, Brody EM. Assessment of older people: self-maintaining and instrumental activities of daily living. Gerontologist. 1969;9(3):179–86. . 5349366

[46] Lyketsos CG, Galik E, Steele C, Steinberg M, Rosenblatt A, Warren A, et al The General Medical Health Rating: a bedside global rating of medical comorbidity in patients with dementia. J Am Geriatr Soc. 1999;47(4):487–91. Epub 1999/04/15. . 10203127 10.1111/j.1532-5415.1999.tb07245.x

[47] Lyketsos C, et al Population-Based Study of Medical Comorbidity in Early Dementia and “Cognitive Impairment, No Dementia (CIND)”: Association With Functional and Cognitive Impairment: The Cache County Study. The American Journal of Geriatric Psychiatry. 2004;13(8):656–64. 10.1176/appi.ajgp.13.8.656 16085781

[48] Sylliaas H, Selbaek G, Bergland A. Do behavioral disturbances predict falls among nursing home residents? Aging Clin Exp Res. 2012;24(3):251–6. Epub 2012/11/02. . 23114551 10.1007/BF03325253

[49] Canevelli M, Adali N, Voisin T, Soto ME, Bruno G, Cesari M, et al Behavioral and psychological subsyndromes in Alzheimer's disease using the Neuropsychiatric Inventory. Int J Geriatr Psychiatry. 2013;28(8):795–803. Epub 2012/11/14. 10.1002/gps.3904 . 23147419

[50] Colombo M, Vitali S, Cairati M, Vaccaro R, Andreoni G, Guaita A. Behavioral and psychotic symptoms of dementia (BPSD) improvements in a special care unit: a factor analysis. Arch Gerontol Geriatr. 2007;44 Suppl 1:113–20. Epub 2007/02/24. 10.1016/j.archger.2007.01.017 . 17317443

[51] Zuidema SU, de Jonghe JF, Verhey FR, Koopmans RT. Neuropsychiatric symptoms in nursing home patients: factor structure invariance of the Dutch nursing home version of the neuropsychiatric inventory in different stages of dementia. Dement Geriatr Cogn Disord. 2007;24(3):169–76. Epub 2007/07/21. 10.1159/000105603 . 17641527

[52] Selbaek G, Engedal K, Benth JS, Bergh S. The course of neuropsychiatric symptoms in nursing-home patients with dementia over a 53-month follow-up period. Int Psychogeriatr. 2013:1–11. Epub 2013/09/26. 10.1017/s1041610213001609 . 24059840

[53] Burgio LD, Park NS, Hardin JM, Sun F. A longitudinal examination of agitation and resident characteristics in the nursing home. The Gerontologist. 2007;47(5):642–9. Epub 2007/11/09. . 17989406 10.1093/geront/47.5.642

[54] Cohen-Mansfield J, Dakheel-Ali M, Marx MS, Thein K, Regier NG. Which unmet needs contribute to behavior problems in persons with advanced dementia? Psychiatry research. 2015;228(1):59–64. Epub 2015/05/03. 10.1016/j.psychres.2015.03.043 ; PubMed Central PMCID: PMCPMC4451402. 25933478 PMC4451402

[55] Cohen-Mansfield J, Thein K, Marx MS. Predictors of the impact of nonpharmacologic interventions for agitation in nursing home residents with advanced dementia. The Journal of clinical psychiatry. 2014;75(7):e666–71. Epub 2014/08/06. 10.4088/JCP.13m08649 . 25093482

[56] Zuidema S, Koopmans R, Verhey F. Prevalence and predictors of neuropsychiatric symptoms in cognitively impaired nursing home patients. Journal of geriatric psychiatry and neurology. 2007;20(1):41–9. Epub 2007/03/08. 10.1177/0891988706292762 . 17341770

[57] Hall GR, Buckwalter KC. Progressively lowered stress threshold: a conceptual model for care of adults with Alzheimer's disease. Archives of psychiatric nursing. 1987;1(6):399–406. Epub 1987/12/01. . 3426250

[58] Smith M, Hall GR, Gerdner L, Buckwalter KC. Application of the Progressively Lowered Stress Threshold Model across the continuum of care. The Nursing clinics of North America. 2006;41(1):57–81, vi. Epub 2006/02/24. 10.1016/j.cnur.2005.09.006 . 16492454

[59] Helvik AS, Engedal K, Wu B, Benth JS, Corazzini K, Roen I, et al Severity of Neuropsychiatric Symptoms in Nursing Home Residents. Dementia and Geriatric Cognitive Disorders EXTRA. 2016;6(1):28–42. Epub 2016/03/05. 10.1159/000442250 ; PubMed Central PMCID: PMCPMC4772643. 26933438 PMC4772643

[60] Cummings JL, McPherson S. Neuropsychiatric assessment of Alzheimer's disease and related dementias. Aging (Milano). 2001;13(3):240–6. Epub 2001/07/11. . 11444257 10.1007/BF03351482

[61] Borza T, Engedal K, Bergh S, Barca ML, Benth JS, Selbaek G. The course of depressive symptoms as measured by the Cornell scale for depression in dementia over 74 months in 1158 nursing home residents. Journal of affective disorders. 2015;175:209–16. Epub 2015/02/02. 10.1016/j.jad.2014.12.053 . 25638794

[62] Carter CL, Resnick EM, Mallampalli M, Kalbarczyk A. Sex and gender differences in Alzheimer's disease: recommendations for future research. Journal of women's health (2002). 2012;21(10):1018–23. Epub 2012/08/25. 10.1089/jwh.2012.3789 . 22917473

[63] Ropacki SA, Jeste DV. Epidemiology of and risk factors for psychosis of Alzheimer's disease: a review of 55 studies published from 1990 to 2003. The American journal of psychiatry. 2005;162(11):2022–30. Epub 2005/11/03. 10.1176/appi.ajp.162.11.2022 . 16263838

[64] Ballard C, Corbett A. Management of neuropsychiatric symptoms in people with dementia. CNS drugs. 2010;24(9):729–39. Epub 2010/09/03. 10.2165/11319240-000000000-00000 . 20806986

[65] Lai CK. The merits and problems of Neuropsychiatric Inventory as an assessment tool in people with dementia and other neurological disorders. Clinical interventions in aging. 2014;9:1051–61. Epub 2014/07/18. 10.2147/CIA.S63504 ; PubMed Central PMCID: PMCPMC4099101. 25031530 PMC4099101

