# Correction: The course of neuropsychiatric symptoms in nursing home residents from admission to 30-month follow-up

**DOI:** 10.1371/journal.pone.0209875

**Published:** 2018-12-20

**Authors:** Anne-Sofie Helvik, Geir Selbæk, Jūratė Šaltytė Benth, Irene Røen, Sverre Bergh

There are some errors in Table 3. The values in the lines "Nighttime Behavior," "Appetite and eating disorder," and "Any symptom" under the section "Cumulative incidence (%) and incidence between two consecutive time points" are incorrect. The corresponding values within the text remain correct. Please see the corrected Table 3 here.

**Table 3:** Prevalence, incidence and persistence of significant neuropsychiatric symptoms (NPS) at each assessment (%).

	T_1_ (Baseline) N = 583	T_2_ (6 months) N = 437	T_3_ (12 months) N = 374	T_4_ (18 months) N = 307	T_5_ (24 months) N = 261	T_6_ (30 months) N = 210
Prevalence						
Delusions	16.0	18.5	19.9	22.3	22.5	21.7
Hallucinations	5.8	8.2	5.0	8.7	10.4	7.6
Agitation	16.2	16.0	21.6	22.3	27.1	25.1
Depression	21.8	22.0	21.6	23.2	21.3	21.6
Anxiety	21.9	20.8	21.2	24.9	26.5	24.1
Euphoria	3.7	4.2	4.6	5.3	6.4	5.9
Apathy	16.6	12.2	13.5	17.5	23.4	21.9
Disinhibition	16.1	16.6	18.5	19.0	29.2	26.6
Irritability	19.2	24.8	27.3	34.5	36.8	37.1
Aberrant Motor Behavior	12.0	11.4	12.1	14.4	15.1	14.0
Nighttime Behavior	17.0	15.0	11.8	12.0	13.9	10.3
Appetite and eating disorder	10.1	9.5	7.4	14.1	12.3	12.3
Any symptom	61.9	55.5	59.5	67.0	69.6	65.4
Cumulative incidence (%) and incidence between two consecutive time points						
	Cumulative incidence (N = 583)	T_1_-T_2_	T_2_-T_3_	T_3_-T_4_	T_4_-T_5_	T_5_-T_6_
Delusions	24.5	13.2	9.5	11.5	12.6	11.4
Hallucinations	10.8	5.8	2.0	4.5	3.7	2.4
Agitation	23.7	9.7	12.6	12.4	12.6	10.5
Depression	22.7	14.4	12.3	14.0	9.8	9.7
Anxiety	24.7	12.4	12.3	13.9	11.9	13.1
Euphoria	9.1	3.2	2.8	4.0	4.9	3.4
Apathy	25.5	7.8	10.4	12.1	15.7	9.7
Disinhibition	24.7	9.0	10.4	11.4	18.0	13.5
Irritability	31.6	15.0	13.6	22.0	15.0	21.0
Aberrant Motor Behavior	17.4	6.7	6.1	9.2	10.0	7.5
Nighttime Behavior	15.8	7.9	4.5	6.4	8.1	4.3
Appetite and eating disorder	23.6	8.9	5.8	12.4	8.3	7.6
Any symptom	28.7	33.7	33.7	47.9	36.5	24.1
Persistence of symptoms at two consecutive time points (%)						
		T_1_-T_2_	T_2_-T_3_	T_3_-T_4_	T_4_-T_5_	T_5_-T_6_
Delusions		46.4	66.7	62.1	52.7	54.3
Hallucinations		48.0	48.0	66.7	75.0	55.6
Agitation		52.3	72.5	57.6	70.7	63.6
Depression		52.3	59.4	57.4	58.8	58.1
Anxiety		52.7	58.8	61.7	67.7	61.7
Euphoria		27.8	31.3	33.3	35.7	54.5
Apathy		35.9	42.9	52.5	51.2	56.5
Disinhibition		53.7	58.2	56.9	63.3	63.0
Irritability		64.1	71.6	67.5	73.6	71.6
Aberrant Motor Behavior		43.4	57.1	56.3	38.9	50.0
Nighttime Behavior		51.4	50.0	45.9	64.0	44.8
Appetite and eating disorder		17.1	40.0	31.8	39.4	45.0
Any symptom		70.1	82.1	80.2	86.3	84.1
